# Pseudo-Continuous Flow FTIR System for Glucose, Fructose and Sucrose Identification in Mid-IR Range

**DOI:** 10.3390/mi9100517

**Published:** 2018-10-13

**Authors:** Hamza Landari, Mourad Roudjane, Younès Messaddeq, Amine Miled

**Affiliations:** 1 LABioTRON Bioengineering Research Laboratory, ECE Department, Université Laval, Québec City, QC G1V 0A6, Canada; hamza.landari.1@ulaval.ca; 2 Research Centre for Advanced Materials (CERMA), Université Laval, Québec City, QC G1V 0A6, Canada; 3 Center for Optics, Photonics and Lasers (COPL), Department of Physics, Université Laval, Québec City, QC G1V 0A6, Canada; mourad.roudjane.1@ulaval.ca (M.R.); Younes.Messaddeq@copl.ulaval.ca (Y.M.)

**Keywords:** pseudo-continuous flow, FTIR spectroscopy, microscopy-FTIR spectrometer, absorption spectrum, Glucose, Fructose, Sucrose, sugars detection and quantification, mid-IR absorption spectroscopy

## Abstract

In this paper, we present a new FTIR-based microfluidic system for Glucose, Fructose and Sucrose detection. The proposed microfluidic system is based on a pseudo-continuous flow coupled to a microscope-FTIR instrument. The detection and characterization of sugar samples were performed by recording their absorption spectrum in the wavelength range 700–1000 cm${}^{-1}$ of the Mid-IR region. The proposed pseudo-continuous flow system is designed to improve the uniformity of the sample distribution in the analyzed area versus conventional systems. The obtained results for different sugars concentrations, show a very low measurement error of 4.35% in the absorption peak intensity, which is ten times lower than the error obtained using the conventional measurements.

## 1. Introduction

Knowing ingredients type and quantity in a consumable food is a highly critical information for safety and population health [1]. Among these ingredients, sugar is a very important element. However, an over consumption of sugar can lead to obesity or health problem related to diabetes [2]. Thus, in food industry, sugar types and quantities are critical for quality control [3]. Consequently, qualitative and quantitative detection of different types of sugar is highly important in the optimization of the production line in the food industry, in the diet for diabetes patients and to reduce the risk of obesity [4,5]. In order to be able to monitor sugar concentration during the production process, several sugar detection methods coupled to a molecular separation techniques were proposed to identify and quantify sugars [6,7,8,9,10,11]. For example, Pettersen et al. used an anion-exchange chromatography process to separate sugar in four different samples of wood and wood pulp hydrolyzed in sulfuric acid [6]. The separated sugar is quantitatively measured by a pulsed amperometric detector. They were able to achieve a limit of detection (LOD) of 13 ng for glucose and 25 ng for arabinose [6]. The main disadvantage of this method is related to the food industry where it needs a very expensive instruments such as chromatography column of sugars molecules and a very sensitive sensor. Also, Jiannong et al. used an electro-chemical detection process with a capillary electrophoresis to separate different types of sugar such as Glucose and Galactose [7]. The achieved LOD was 1 fMol for both Glucose and Galactose with a relative standard deviation of 5% [7]. For this method, contamination risk is very high where a molecular oxidation can take place, which leads to a change in the molecular charge density. An optical sensing method was proposed by Roig and Thoms, to extracte sugar concentration (Glucose, Fructose, Sucrose and Lactose) from fruit juices and soft drinks using ultraviolet (UV) photo-degradation and UV spectrophotometry process (UV/UV) [8]. This extraction method was inspired from the UV/UV process developed for the measurement of some wastewater pollution parameters [12,13]. They were able to detect sugar concentration ranging from 40 g/L to 500 g/L. Unlike other methods, this technique has a low risk of contamination but the achieved LOD with this setup remains very high for sugars quantification in commercial food. Based on the same principle of optical sensing, Duarte et al. used the Fourier Transform InfraRed spectroscopy (FTIR) with wavelength ranging from 900 cm${}^{-1}$ to 1250 cm${}^{-1}$ coupled to an Attenuated Total Reflectance (ATR) cell, in order to detect the ripening of Mango through the quantification of the sugar concentration in Mango juice [9]. The achieved LOD was 1.5 g/L with a prediction error of 4.9%. The achieved LOD with the ATR-FTIR method is very high as in the case of the UV/UV method [8]. Also, the experimental setup requires dedicated and expensive equipments such as a crystal for the ATR cell which is not compatible with all types of solvents. Several other researchers developed other sugar detection techniques using FTIR spectrometer coupled to different sample handling systems such as (ATR) cell [10,14] and Partial Least Squares (PLS) [11]. The optical detection methods remain among the best approaches for sugar detection, particularly in commercial production. Indeed, optical sensing is a contactless detection technique and thus, it enhances the quality control and reduce contamination risk. The FTIR spectrometer is a well-used equipment in a lot of chemistry and physics experiments, such as quantitative analysis of complex mixtures [15,16], investigation of dynamic systems [17,18], biological and biomedical analysis [19,20,21,22], micro spectroscopy and hyper spectral imaging [23,24], and in the study of many types of interfacial phenomena [25,26].

**Figure 1:**
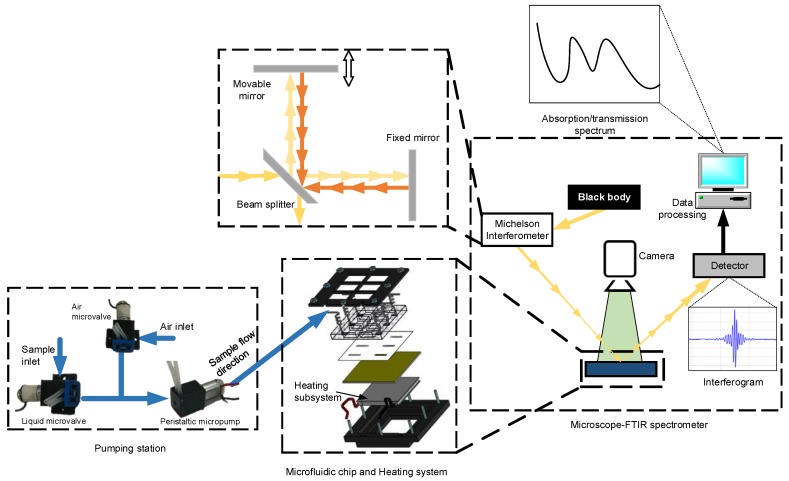
Schematic representation of the working principle of the proposed technique for sugar detection coupled to the developed microfluidic chip for sampling and sample handling and temperature control.

Indeed, the FTIR spectroscopy is a powerful tool to detect solid, liquid and gas phase species through their absorption spectrum in a wide wavelength range. The operating principle of FTIR spectrometer is mainly based on (i) the illumination of the sample with a multiple wavelength light beam, (ii) the measurement of its absorption and (iii) a data processing system. The used beam light is usually generated from a broadband light source (often a black body), and transmitted through a Michelson interferometer in order to excite the sample with separate wavelength. As shown in Figure 1, the operating principle of this interferometer is based on a beam splitter that splits the beam of light, and two reflective mirrors. The movement of one of the reflective mirrors is monitored by a servomotor in order to control the light path-length and then creates an optical interference between the two divided light beams, which generates a specific interferogram [27]. The sample handling system depends mainly on the analyzed sample types (solid, liquid or gaseous) and their quantities. When a microscope is coupled to an FTIR spectrometer as a sample handling system, it can be used in polymer, fiber, pharmaceuticals, semiconductors, biochemistry, and chemical analysis [28]. The main advantage of the microscope resides in the fact that it can be used for the analyses of a small sample droplet and evaluate its composition and molecular distribution. In this case, the microscope requires a sample handling support (SHS) to hold the sample droplet.

Recently, we have used a microscope-FTIR setup with a standard SHS made of a total reflective mirror (TRM) to perform a quantitative and a qualitative detection of sugars in artificial solutions. However, these solutions contain an important quantity of water which is opaque in the Mid-IR region. Thus, water solvent must be replaced with other liquid or evaporated. Unfortunately, most solvents that transmit in this region of wavelengths are toxic and have a very low solubility such as methanol [29]. Though water evaporation is more suitable for sugar detection in aqueous solution. Despite the solvent evaporation, it was observed that the sugar sample is not uniformly deposited on the TRM. To address this problem, and enhance repeatability of the analysis, we designed a new SHS.

In this paper, a new microfluidic device for microscope-FTIR based sugar sensing in Mid-IR is presented. The main advantage of the designed microfluidic device is its ability to generate a uniform thin layers of sugar from a liquid sample. The proposed experimental setup used for sugar detection is shown in Figure 1. It consists of 3 main parts: (1) pumping subsystems (2) microscope-FTIR spectrometer, and (3) microfluidic chip with a heating system.

This paper is organized as follows, Section 2 describes the developed system for sugar detection and its different components, the experimental setup for sugar detection is presented in Section 3. Finally the results of several analyzed samples of synthetic Glucose, Fructose and Sucrose are reported and discussed in Section 4.

## 2. Microfluidic System Description

**Figure 2:**
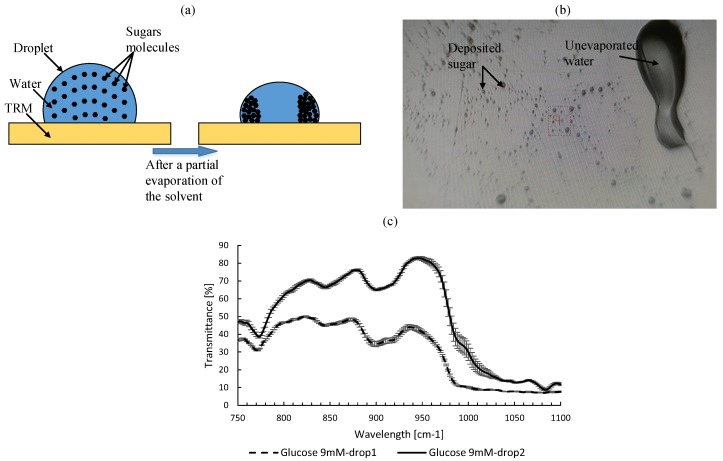
(**a**) Schematic representation of the difference between the molecular distribution in the droplet before and during the solvent evaporation using the standard SHS. (**b**) Deposited sugar on a standard SHS after water evaporation. (**c**) Two recorded absorption spectrum of two sugar samples taken from the same sugar solution.

When we use the standard SHS for sugar detection, a droplet sample is deposited on the gold surface of the TRM using a micro-pipette. The solvent is then evaporated naturally, and consequently a non-uniform sugar layer is deposited on the TRM surface. The main disadvantages of this setup are: (i) the sugars are not deposited in the same position where the optical detector is calibrated, i.e., where the image background was acquired; (ii) the time needed to evaporate the solvent is very long (more than 30 min), which leads to a non-uniform distribution of sugar molecules density inside the droplet because of the gravity force, and surface tension that attracts molecules to the edges of the droplet. Figure 2a,b shows how sugars are deposited using the standard SHS, and the molecular distribution in the droplet during the solvent evaporation, respectively. In the case of a non-uniform distribution, two samples with the same concentration were analyzed with the standard TRM, and a high deviation (from 41.6% to 224.6% depending on the wavelength) is observed between the two spectra as shown in Figure 2c. Thus, the recorded absorption spectra are not reproducible, and the results of analysis are not reliable though.

**Figure 3:**
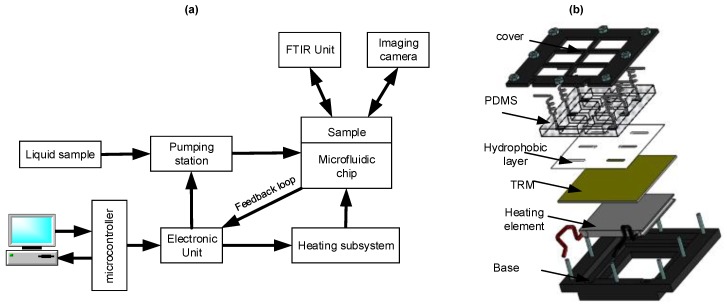
(**a**) Functional diagram of the entire system. (**b**) Microfluidic chip with heating subsystem.

To overcome this problem and to achieve a better and uniform sugar distribution layer, the liquid sample is injected using a pseudo-continuous flow device into the analysis area. Then, the solvent is evaporated and a thin layer of sugar is deposed on the analysis area. The functional diagram of the entire device is shown in Figure 3a. The developed SHS includes a subsystem to heat the analysis area, a microfluidic chip which is made with a TRM, a Polydimethylsiloxane (PDMS) chamber for liquid confinement, and a hydrophobic vinyl layer. Flow control is achieved through a designed pumping station controlled by an electronic unit.

The TRM is designed and fabricated in our laboratory by a deposition of a thin gold layer with a thickness of 150 nm on the upper surface of a 50 mm × 50 mm glass slide, using a physical evaporation technique. This gold layer ensures a highly reflective surface of the TRM. The latter was cleaned in an ultrasonic bath with acetone in order to remove any residual contamination on the gold surface. A separate 50 μm vinyl layer is treated with a hydrophobic spray (Ultrahydrophobic Spray, Waterbeader, Dalton, GA, USA), and then glued on the gold surface (the upper face of the TRM). Circular spots with rectangular fluidic open micro-channels are designed on the vinyl layer to form the analysis area, and to control the flow direction, respectively. The surface treatment of the vinyl layer is performed to ensure that the injected sample remains in the analysis area, and no overflow of liquid will take place. Finally, a PDMS substrate with square chambers is mounted on the top of the vinyl hydrophobic layer to (i) separate each analysis site in order to eliminate the risk of cross contamination between nearby analysis area, (ii) to ensure microfluidic connections, and (iii) to guide the liquid and airflow through a printed micro-channels on its lower side.

**Figure 4:**
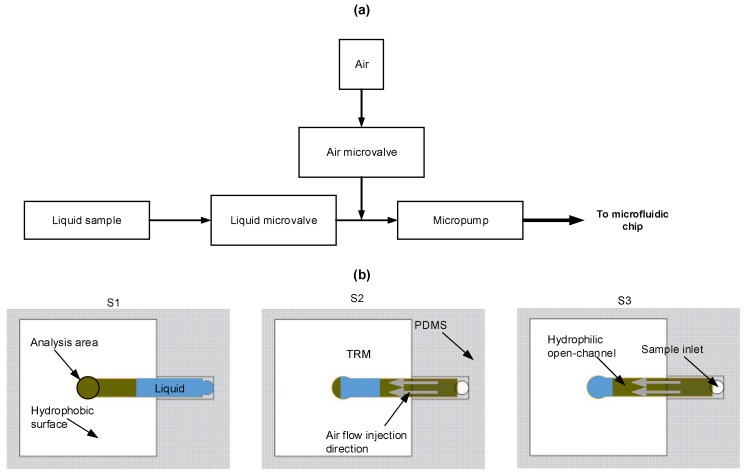
(**a**) Pumping unit diagram. (**b**) Microfluidic sample handling steps: (S1) is the initial step where the liquid is injected; (S2) is the second step where the liquid is sampled by air injection; and (S3) is the last step where air is continuously injected to keep the liquid far from the PDMS chamber walls during the solvent evaporation.

The sample injection is ensured by a pumping unit which consists of a peristaltic micropump and two microvalves as shown in Figure 4a. To avoid overflow of the liquid during injection, the micropump flow rate was set to 0.43 mL/min. Indeed, with a higher flow rate, the liquid can quickly leave the open micro-channels. Microvalves were used to control the quantity of injected liquid. 120 μL of each analyzed sample is injected in a separate hydrophilic site. The volume of injected sample is controlled through the opening duration of the liquid-microvalve (an opening of 17 s is needed to generate a volume sample of 120 μL). Once the hydrophilic spot is filled, the liquid-microvalve is closed and the air-microvalve is open. Then, the micropump generates a continuous and a constant air flow rate through the micro-channel until the solvent reaches the hydrophilic spot (analysis area), where a sugar layer is deposed after solvent evaporation. The air injection into the microfluidic chip is a very important step, otherwise no sugar layer will be deposited in the analysis area due to the PDMS chamber wall attraction of the liquid sample, and the sugar will be only deposited close to the walls of the PDMS micro-channel. Figure 4b shows the different steps of the sample-handling process.

The solvent evaporation is ensured by a heating subsystem made with a 40 mm × 40 mm × 3.9 mm Peltier module (TEC1-12706, Hebeiltd, Shanghai, China) which was attached to the back side of the TRM using a thermal paste (ARCTIC, MX-4, Hong Kong, China) that guarantee the uniform propagation of the heat from the Peltier module to the TRM surface. The power consumption of the heating subsystem is 5 W which is sufficient to reach 80 ${}^{\circ }$C on the TRM upper side.

**Figure 5:**
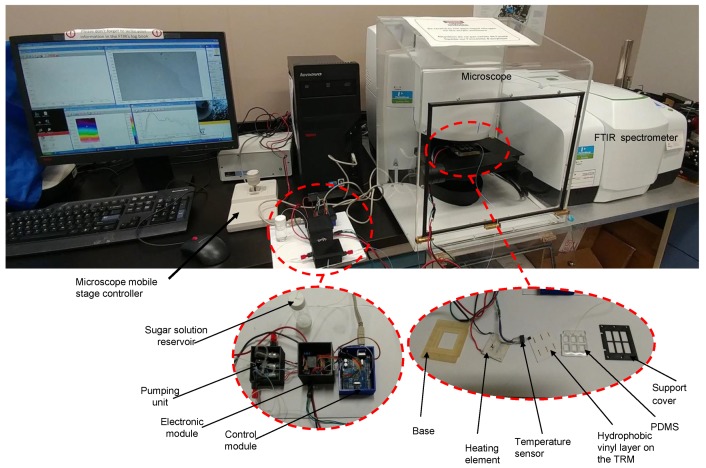
Experimental setup for sugar identification and quantification based on a microscope-FTIR spectrometer with the new designed microfluidic device.

The micropump, the microvalves and the Peltier modules are controlled by a unique electronic unit. It is essentially composed of a temperature sensor (LM35, Texas Instruments, Dallas, TX, USA) placed on the upper side of TRM for the feedback loop between the heating subsystem and the electronic control unit. A MOSFET transistor (VN2222, Microchip Technology, Chandler, AR, USA), and a relay (G5LA-1A4 DC5, Omron Electronics, Shiokoji Horikawa, Shimogyo-ku, Kyoto, Japan) are needed to turn ON/OFF the heating subsystem and monitor the pumping unit. They are also embedded in the electronic unit. A micro-controller (ATmega, ATMEL, San Jose, CA, USA), with a serial interface (RS232) to send/receive data to/from computers, is used to control the electronic unit, to monitor the developed device and to acquire the user configuration such as temperature and sample volume. In Figure 5 we show the entire experimental set up for sugar identification/quantification with the microscope FTIR spectrometer coupled to the new designed microfluidic chip.

**Figure 6:**
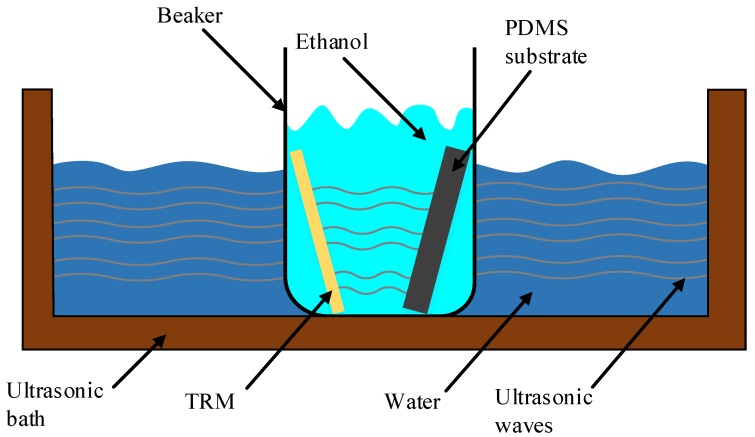
Cleaning setup of the TRM and the PDMS substrate.

The TRM and the PDMS subtract are reused after a cleaning process. This process consists of the use of an ultrasonic bath with water and a beaker filled with ethanol. Then, the TRM and the PDMS subtracts are emerged for 30 min inside the bath. This cleaning method, shown in Figure 6, is used to remove all the residue of the sugars from the gold surface and PDMS chamber walls. In addition, the microfluidic tubing is cleaned by two successive injections of ethanol and deionized water respectively.

## 3. Experimental Procedure

**Figure 7:**
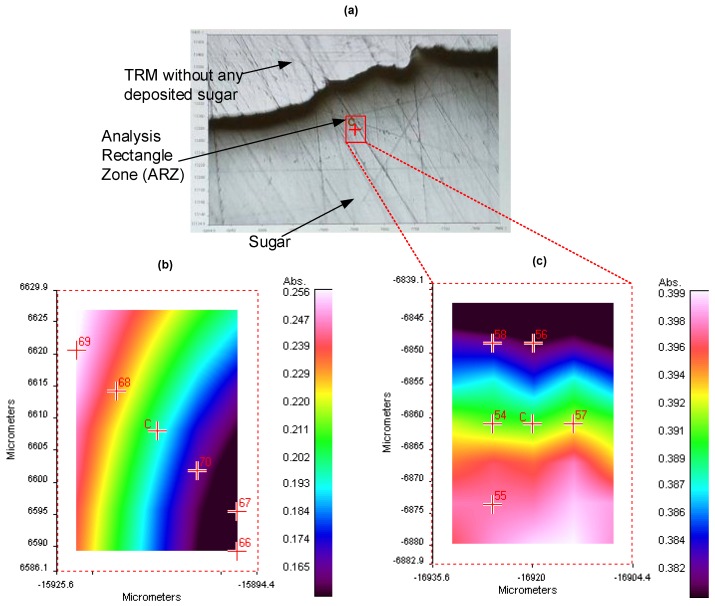
(**a**) Sugar layer deposited on the hydrophilic surface of the TRM. The 2D graphs of sugar absorption obtained in the ARZ using (**b**) the classical TRM, and (**c**) the new microfluidic device. The variation of the absorption intensity is shown in the right column for each 2D graph.

In this section we describe our experimental procedure to detect and quantify sugars using our new SHS. Glucose (SIGMA-G8270, Sigma-Aldrich, Saint Louis, MT, USA), Fructose (SIGMA-F2543, Sigma-Aldrich, Saint Louis, MT, USA) and Sucrose (SIGMA-S7903, Sigma-Aldrich, Saint Louis, MT, USA) sugars were used without any purification. Liquid solutions were prepared with different concentrations (1 mM, 3 mM, 5 mM, 10 mMol and 20 mMol) for each sugar type using deionized water as a solvent. 120 μL of sugar sample is injected into the analysis area by the peristaltic micropump through the micro-channel. Then, the TRM surface is heated with the Peltier module during 3 min to reach 80 ${}^{\circ }$C. When the solvent is completely evaporated, a thin layer of sugar is deposited on the hydrophilic spot. Finally, this thin layer is cooled down during 5 min to reach 35 ${}^{\circ }$C. The sugar identification and quantification are achieved based on their absorption spectra recorded using microscope-FTIR spectrometer (Spotlight 400 FT-IR Imaging System, Perkinelmer, Waltham, MI, USA) in the wavelengths range from 750 cm${}^{-1}$ to 1000 cm${}^{-1}$ with a resolution of 4 cm${}^{-1}$ and 32 scans average. First, a fixed Analysis Rectangle Zone (ARZ) (43 μm × 31.2 μm) of the sugar layer is set by the users as shown in Figure 7a. Within the ARZ, the microscope scans 70 different sub-zones for each absorption wavelength. The displacement between different analyzed sub-zones is ensured by the mobile stage of the microscope with a resolution of 6.25 μm. Obtained results are reported in a 2D graph in Figure 7b,c. The 2D graph reflects the quality of the deposited sugar distribution in the ARZ. Prior to sample injection, the background absorption spectrum is recorded. It represents the measured intensity of absorbed light by the bare TRM gold surface that needs to be subtracted from all sugar spectra.

The final absorption spectrum of each sugar sample is an average over 4 spectra recorded at four different sub-zones of the ARZ. The sample analysis is repeated 3 times for each concentration in order to verify the repeatability of the results.

## 4. Results and Discussion

### 4.1. System Optimization

**Figure 8:**
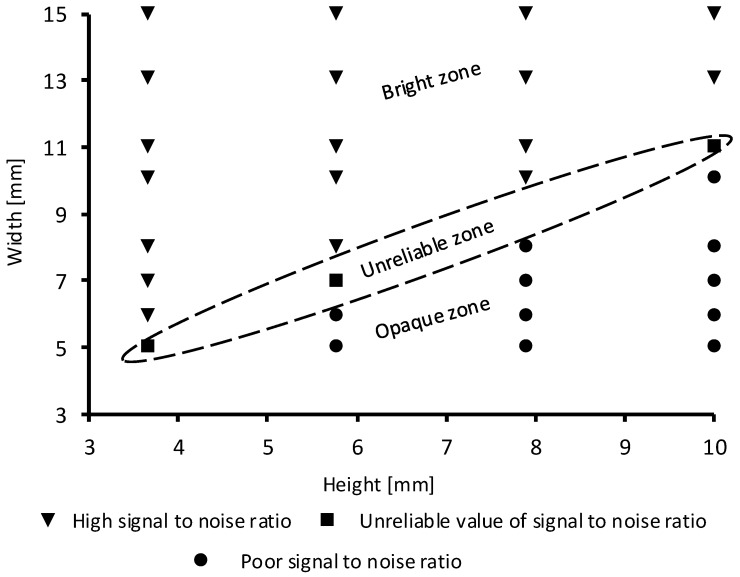
Tested height-width combination of the PDMS chambers. Three zones are identified: Bright, opaque and unreliable zones.

The major source of noise in our experiment comes from the possible interaction of the Mid-IR beam light with the surrounding materials (the vinyl layer and the PDMS chamber walls) before it reaches the deposited sugar. To minimize this interaction, the PDMS chamber and the hydrophilic spot must be large enough so that the beam reflection occurs only on the TRM without any interaction with neither the PDMS chamber walls nor the spot edges. Therefore, an optimization of the architecture of the microfluidic system is required to reduce the noise effect. Several spots of the hydrophobic vinyl layer with different diameters (0.5 mm, 1 mm, 2 mm and 3 mm) were designed. The obtained results show that for a spot diameter less than 2 mm the absorption spectrum of sugar is indistinguishable from noise, i.e., Glucose peaks are not clearly identified, for example. Using the optimized spot size (2 mm) of the vinyl layer, 32 different PDMS chambers with different heights (4 mm, 6 mm, 8 mm and 10 mm) and width (5 mm, 6 mm, 7 mm, 8 mm, 10 mm, 11 mm, 13 mm and 15 mm) were fabricated and tested as shown in Figure 8. In this figure, it can be seen that the dimensions are interdependent. Indeed, an increase in the chamber height must be compensated by an increase in its width in order to eliminate the interaction between the beam of light and the PDMS chamber walls. As shown in Figure 8, depending on the height-width of PDMS chamber, three zones are identified : (1) An opaque zone where sugar peaks cannot be identified; (2) a bright zone where sugar can be well identified and quantified; finally (3) an unreliable zone where the recorded absorption spectrum of sugar is not reproducible within the same ARZ for the same concentration of sugar. Based on this study, the minimum dimension of the PDMS chamber corresponds to 6 mm width and 4 mm height.

In the case of our experiment, the dimensions of the microfluidic device were fixed to 2 mm for hydrophilic spot diameter and 12 mm × 4 mm for the width × the height of the PDMS chamber.

Sugar distribution profiles were acquired using the standard SHS and the designed microfluidic chip mounted separately on the microscope-FTIR setup. Based on the results reported in Figure 7b, a variation of 43.23% of the absorption intensity is measured within the ARZ using the standard SHS. This high variation is due to the non-uniform profile of the sugar distribution. But, as it can be seen in Figure 7c, the variation of the absorption intensity using our designed system is 10 times smaller (4.35%) than the standard one, which proves that the designed microfluidic chip provides a better uniformity of the distribution of the deposited sugar.

### 4.2. Qualitative Detection

**Figure 9:**
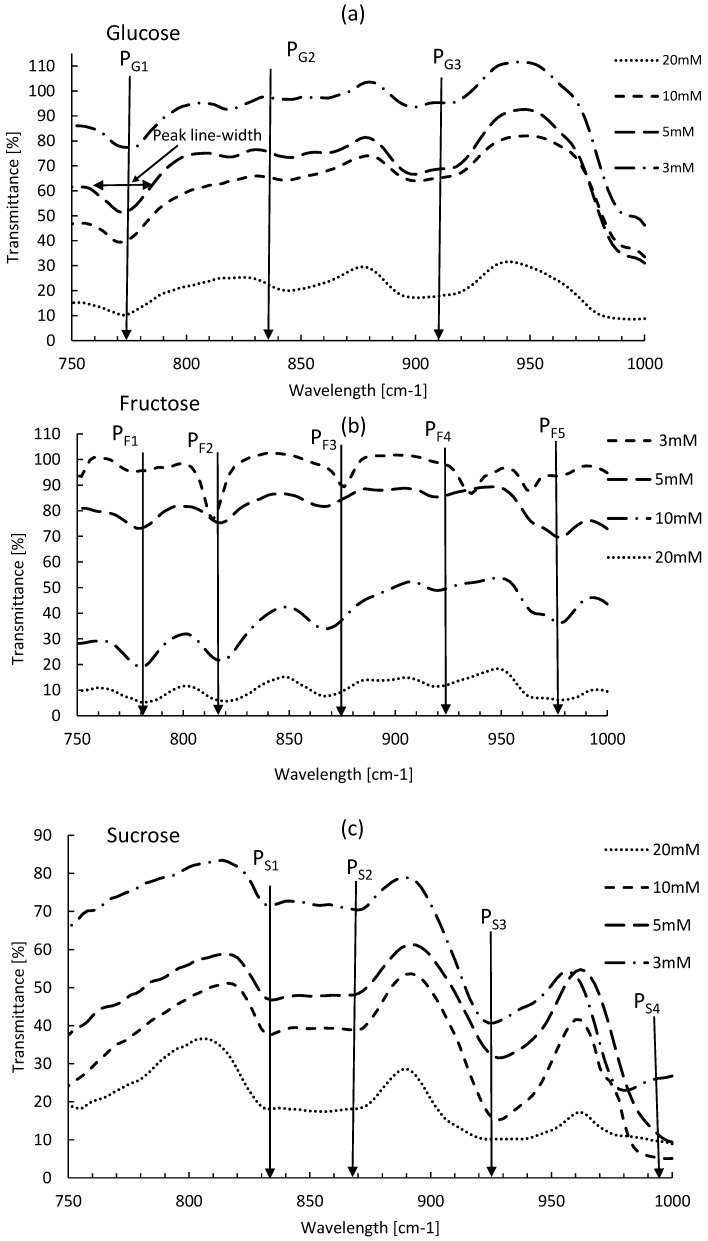
Transmission spectra for different sugar types at different concentrations. (**a**) Glucose spectra; (**b**) Fructose spectra; (**c**) Sucrose spectra.

The absorption spectra of Glucose, Fructose and Sucrose are recorded for different concentrations, and reported in Figure 9 as a transmitted signal (transmittance %). The obtained absorption spectra for each sugar are in a good agreement with the literature [29,30]. In Figure 9a, Glucose fingerprint is composed of three absorption peaks located at 774 cm${}^{-1}$ ($P_{G1}$), 834 cm${}^{-1}$ ($P_{G2}$) and 910 cm${}^{-1}$ ($P_{G3}$). In the case of the Fructose, as it is shown in Figure 9b, the obtained absorption spectrum contains five different peaks detected at 784 cm${}^{-1}$ ($P_{F1}$), 818 cm${}^{-1}$ ($P_{F2}$), 870 cm${}^{-1}$ ($P_{F3}$), 924 cm${}^{-1}$ ($P_{F4}$) and 978 cm${}^{-1}$ ($P_{F5}$). For the Sucrose, the absorption spectrum shown in Figure 9c has four peaks, measured at 832 cm${}^{-1}$ ($P_{S1}$), 868 cm${}^{-1}$ ($P_{S2}$), 922 cm${}^{-1}$ ($P_{S3}$) and 992 cm${}^{-1}$ ($P_{S4}$). However, the peak at 832 cm${}^{-1}$ was not observed in literature [29]. Therefore, it could be related to an impurity or other interfering molecule in our Sucrose sample. Thus, this peak will not be used for Sucrose identification/quantification.

Based on the unsaturated absorption spectrum reported in Figure 9, we have measured for each absorption peak the corresponding line-width (L-W), which is defined as the full width at half maximum. Obtained results are summarized in Table 1.

Based on our experiments, we have investigated the LOD for each sugar. This LOD represents the minimum sugar concentration that can be detected with our proposed detection system. Below this LOD threshold, the deposited sugar layer on the analysis hydrophilic spot after evaporation is not sufficient enough to provide an absorption spectrum for a sugar identification and quantification. The LOD obtained in this work is 3 mm for both, Glucose and Fructose and 1 mm for Sucrose.

### 4.3. System Calibration and Quantitative Analysis

An empirical method was developed to calibrate the new developed system and perform quantitative detection of different sugars. It is based on the relation between the peak absorption intensity and the sugar concentration. Several curves were plotted for each sugar molecule, reporting the sugar concentration as a function of the absorption intensity for each detected peak. Quadratic and linear equations are used to fit the data. The best fit is obtained for the highest $R^{2}$. For all sugars, the results of quadratic and linear fitting parameters are reported in Table 2 and Table 3, respectively. The empirical calibration equations are selected based on two criteria: (1) the absorption peak must be well isolated, not saturated, with the narrowest line-width; (2) the $R^{2}$ coefficient must be the highest one among the others (ideally close to 1). For all sugar concentrations, the reported absorption intensity is the average value over the absorption intensities of three tested samples. Though for each reported intensity value, an error bar is drawn on the plotted curve, which corresponds to the standard deviation (σ).

#### 4.3.1. Glucose Calibration Equation

**Figure 10:**
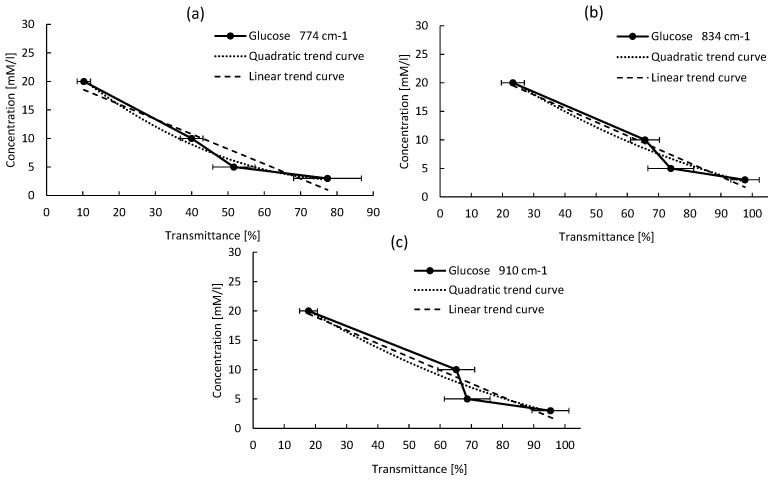
Glucose absorption intensity versus its concentration for different peaks, for the absorption peaks at (**a**) 774 cm${}^{-1}$, (**b**) 834 cm${}^{-1}$, (**c**) 910 cm${}^{-1}$.

As shown in Figure 10, for most Glucose absorption peaks, both linear and quadratic equations fit well the variation trends with a high $R^{2}$ coefficients. While the linear fit is expected to give a better prediction of the variation of the concentration as a function of the absorption intensity compared to the quadratic fit, the results of analysis listed in Table 2 and Table 3, show that the quadratic fit is better than the linear fit based on the $R^{2}$ coefficients. A possible explanation of this result could be related to the uniformity of the sugar layers formed on the TRM surface, which could be locally inhomogeneous though. Consequently, the quadratic parameters are used to determine the calibration equation. If we consider only the second criteria, Glucose concentration can be extracted from any one of its four representative peaks. Although the peak located at 834 cm${}^{-1}$ has the highest $R^{2}$ coefficient (see Table 3), its line-width is very broad though for all concentrations lower than 20 mm. Consequently, it cannot be used for Glucose quantification based on the first criteria. From this study, the absorption peak located at 774 cm${}^{-1}$ (L-W = 22 cm${}^{-1}$ and $R^{2}$ = 0.9864) is the one that fulfills the two criteria conditions, and thus it is selected as the most relevant peak.

#### 4.3.2. Fructose Calibration Equation

**Figure 11:**
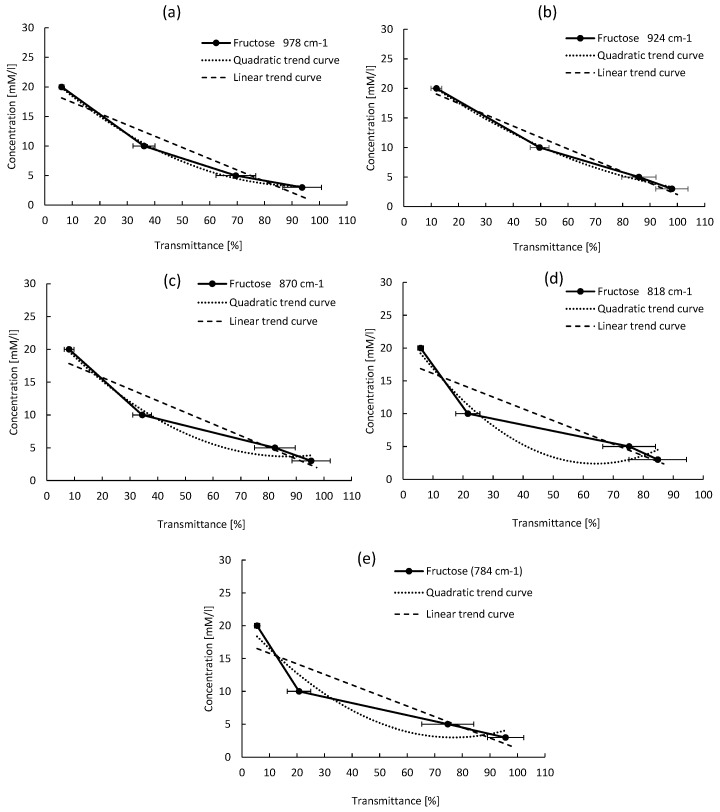
Fructose absorption intensity as a function of its concentration for different peaks, for the absorption peaks at (**a**) 978 cm${}^{-1}$, (**b**) 924 cm${}^{-1}$, (**c**) 870 cm${}^{-1}$, (**d**) 818 cm${}^{-1}$, (**e**) 784 cm${}^{-1}$.

For the five absorption peaks of Fructose, the quadratic and linear fits are shown in Figure 11. From this figure, the variation trends are well predicted with the first and second order equations for only the peak located at 818 cm${}^{-1}$ ($P_{F2}$), versus the other peaks. However, like in the case of Glucose analysis, based on the $R^{2}$ coefficient values listed in Table 2 and Table 3, the quadratic equation has a better prediction of the concentration variation as a function of the absorption intensity for the peak located at 818 cm${}^{-1}$ in comparison to the linear fit. Based on the selection criteria of representative peaks, this peak is the one that fulfills these conditions because it has the narrowest L-W = 25 cm${}^{-1}$, and a very high $R^{2}$ = 0.9495 coefficient.

#### 4.3.3. Sucrose Calibration Equation

**Figure 12:**
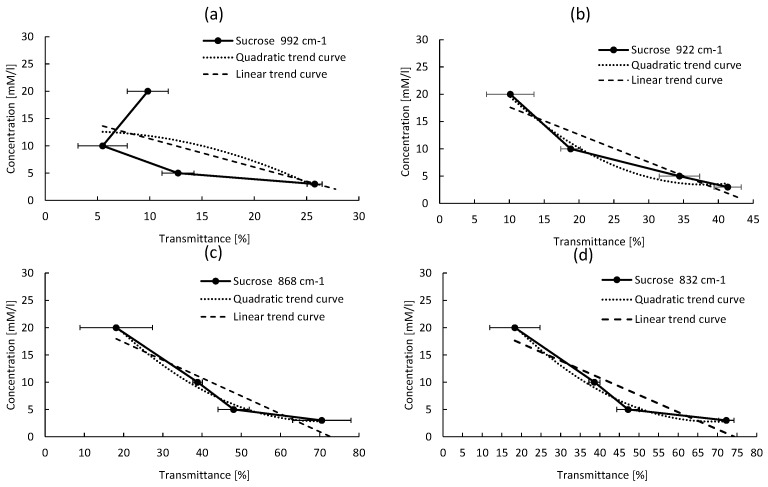
Sucrose absorption intensity versus its concentration for different peaks, for the absorption peaks at (**a**) 992 cm${}^{-1}$, (**b**) 922 cm${}^{-1}$, (**c**) 868 cm${}^{-1}$, (**d**) 832 cm${}^{-1}$.

Finally, for Sucrose, we found that the quadratic equation has a better fitting of the variation trends, like in the case of Glucose and Fructose. Obtained results are reported in Figure 12. From this analysis and based on the same criteria described in Section 4.3, the peak located at 922 cm${}^{-1}$ ($P_{S3}$) is chosen to quantify Sucrose. This peak has the higher $R^{2}$ coefficient (0.9796) and a narrower L-W (38 cm${}^{-1}$) compared to other peaks shown in Figure 12.

After calibrating the detection setup, and in order to validate sugar quantification with the proposed system, Glucose, Fructose and Sucrose sugar solutions with unknown concentration were analyzed. Their absorption spectrum was recorded as described above, and the concentration is calculated from the absorption intensities of their characteristic peaks using the empirical equations, and results are listed in Table 2. From this table, two Glucose sugar concentrations were calculated at 6.64 mm and 14.80 mm, while the initial concentration was 7 mM and 15 mM, which corresponds to an identification error of 5.1% and 1.3%, respectively. In the case of Fructose, the identification errors of two sugar concentrations were calculated at 1.41% and 1.68%, while the identification errors of two Sucrose concentrations were calculated at 7.9% and 3.3%. The quantification of sugar concentrations using the linear fit has led to a higher identification error as shown in Table 3, which confirms that the quadratic analysis provides promising results using our system.

#### 4.3.4. Discussion

Microscope-FTIR spectrometer is a well established qualitative measurement technique [28]. However, using the standard microscope-FTIR for measurements without our SHS did not allow a reliable quantitative measurement. This is mainly due to the non-uniformity of the collected data when a manual liquid handling approach is used (pipette). Indeed, as shown in Figure 7, the non-uniformity of the analyzed samples is improved from 43.23% to 4.35% with our proposed SHS. Technically speaking, it is not possible to control the liquid distribution uniformity manually. Indeed, with our SHS, we control the liquid sample shape and volume because the designed hydrophobic surface pushes the molecules to be confined in the sampling region only. Also the microfluidic system ensures a very accurate volume control as we are using peristaltic micropump with optimized motor for accurate microfluidic control. The final objective is to reach a linear approximation between the absorption intensity and the concentration at a well defined absorption peak of sugar to establish a standard curve in order to estimate sugars concentration. However, the linear approximation does not allow precise estimation of the concentration as shown in Table 3 compared to quadratic approximation. For example, using our quadratic approximation approach, the maximal error in Glucose is 5.1% at 774 cm${}^{-1}$ wavelength while it is 18.9% with linear approximation.

Consequently, our work is mainly focused on the automation of the system and automated sugar identification. Also, our proposed system addressed sample non-uniformity issue to quantify the sugar concentration. It is worth noting that the achieved LOD in this study is mainly related to the microscope-FTIR itself which is a commercial system. Therefore, in this work our main interest is to ensure a reliability of the results as the approach is dedicated for food industry. One of the main challenges in this industry is how to provide a reliable analysis technique. Thus, the uniformity of the deposited thin layer of sugar is very critical in this case of study.

Finally, the system at this stage assumes that the liquid solution has been pretreated so that it contains only one type of sugar, thus, presented results are limited to homogeneous solution.

## 5. Conclusions

In this paper, a microscope-FTIR spectrometer was used for Glucose, Fructose and Sucrose detection in Mid-IR using a new microfluidic system for sample handling. The system is mainly composed of a liquid sampling and sample handling device with a heating subsystem to evaporate the solvent of the liquid sample. A hydrophobic layer and a PDMS chamber were also used to ensure a uniform sample distribution of deposited sugar sample on the analysis area after water evaporation. The main objective of our developed system is to enhance the sugar detection repeatability using the microscope-FTIR and consequently, to identify and quantify sugar types in aqueous solution. With the new microfluidic system, we have demonstrated that the variation on the absorption intensity of sugar molecules in the layers did not exceed 5%, while it goes further than 30% with a conventional setup. This small variation is directly related to the uniformity of the molecular distribution in the thin sugar layers. This uniformity was achieved by two approaches: (1) a controlled pseudo-continuous flow of the injected sample injected into the analysis area; and (2) a uniform evaporation of the solvent to form an homogeneous sugar layers. We also developed an empirical method to calibrate our experimental set up, and to estimate the concentration of different sugars. This method is based on a quadratic fit of the variation of the absorption intensity as a function of the concentration for Glucose, Fructose, and Sucrose. We observed that the quadratic fit reproduces the variation trends for the three sugars in a better way. Using our system, we were able to quantify an unknown sugar concentration with a maximum error of 5.1% for Glucose, 1.68% for Fructose, and 7.9% for Sucrose.

## Figures and Tables

**Table 1:** Absorption peak signatures (fingerprint) of Glucose, Fructose, and Sucrose, the line-width of each characteristic peak and sugars LOD of microscope-FTIR spectroscopy coupled to our designed microfluidic chip.

Sugars Type	Peak Position (cm${}^{-1}$)	Reported Peaks Positions ${}^{⋆⋆}$ (cm${}^{-1}$)	Position Error (cm${}^{-1}$)	L-W (cm${}^{-1}$)	LOD (mm)
Glucose	774 ($P_{G1}$)	776	3	22	
	834 ($P_{G2}$)	836	4	Weak	3
	910 ($P_{G3}$)	915	6	40	
Fructose	784 ($P_{F1}$)	780	2	27	
	818 ($P_{F2}$)	818	2	25	
	870 ($P_{F3}$)	873	3	32	3
	924 ($P_{F4}$)	923	4	Weak and Diffuse	
	978 ($P_{F5}$)	977	2	35	
Sucrose	832 ($P_{S1}$)	834	7	Blend ${}^{⋆}$	
	868 ($P_{S2}$)	869	7	Blend ${}^{⋆}$	
	922 ($P_{S3}$)	924	3	38	1
	992 ($P_{S4}$)	994	6	Blend ${}^{⋆}$	

${}^{⋆}$ Absorption peak is so large causing an overlap between close peaks. ${}^{⋆⋆}$ Reported values in [29,30].

**Table 2:** Absorption peaks characteristics and quantification results of unknown solution concentration based on quadratic equation.

Sugars Type	Absorption Peaks Positions (cm${}^{-1}$)	Empirical Function	$R^{2}$	Measured Absorption Intensity (%)	Calculated Concentration (mm)	Real Concentration (mm)	Quantification Error (%)
	**774($P_{G1}$)**	**[C] = 0.003$T^{2}$ − 0.537T + 25.353**	**0.9864**	**49.300**	**6.644**	**7**	**5.1**
				**22.690**	**14.808**	**15**	**1.3**
Glucose	834 ($P_{G2}$)	$\left[C\right]=0.001T^{2}-0.388T+28.470$	0.9686	77.960	6.076	7	13.2
				40.945	14.738	15	1.7
	910 ($P_{G3}$)	$\left[C\right]=0.001T^{2}-0.351T+25.940$	0.9462	78.216	5.200	7	25.7
				24.416	18.021	15	20.1
	784 ($P_{F1}$)	$\left[C\right]=0.003T^{2}-0.463T+20.833$	0.9187	35.199	8.221	7	17.4
				15.100	14.512	15	3.2
	**818 ($P_{F2}$)**	**[C]** **=** **0.005** $T^{2}$ **−** **0.633** T **+ 22.704**	**0.9495**	**33.093**	**7.099**	**7**	**1.41**
				**13.081**	**15.253**	**15**	**1.68**
Fructose	870 ($P_{F3}$)	$\left[C\right]=0.002T^{2}-0.4457T+23.065$	0.9804	20.458	15.034	7	114
				40.426	9.296	15	38
	924 ($P_{F4}$)	$\left[C\right]=0.001T^{2}-0.337T+23.76$	0.9974	45.789	11.031	7	57.6
				23.81	16.461	15	9.741
	978 ($P_{F5}$)	$\left[C\right]=0.002T^{2}-0.404T+22.237$	0.9971	31.523	11.572	7	65.3
				2.881	21.088	15	40.6
	832 ($P_{S1}$)	$\left[C\right]=0.006T^{2}-0.941T+35.134$	0.9886	39.063	8.732	7	24.7
				35.090	10.466	15	30.2
	868 ($P_{S2}$)	$\left[C\right]=0.006T^{2}-0.904T+34.431$	0.9897	41.083	8.234	7	17.6
Sucrose				33.482	11.426	15	23.8
	**922** **(** $P_{S3}$ **)**	[C] = **0.020**$T^{2}$ − **1.566**T + **33.262**	**0.9796**	**23.976**	**7.555**	**7**	**7.9**
				**14.902**	**14.498**	**15**	**3.3**
	992 ($P_{S4}$)	$\left[C\right]=-0.020T^{2}+0.148T+12.366$	0.3727	7.765	12.285	7	75.5
				5.623	12.554	15	16.3

**Table 3:** Absorption peaks characteristics and quantification results of unknown solution concentration based on linear equation.

Sugars Type	Absorption Peaks Positions (cm${}^{-1}$)	Empirical Function	$R^{2}$	Measured Absorption Intensity (%)	Calculated Concentration (mm)	Real Concentration (mm)	Quantification Error (%)
	774 ($P_{G1}$)	[C]=−0.2614T+21.212	0.9175	49.3	8.325	7	18.9
				22.69	15.281	15	1.9
Glucose	834 ($P_{G2}$)	[C]=−0.2391T+25.07	0.9534	77.96	6.429	7	8.2
				40.945	15.28	15	1.9
	910 ($P_{G3}$)	[C]=−0.2273T+23.532	0.9321	78.216	5.753	7	17.8
				24.416	17.982	15	19.9
	784 ($P_{F1}$)	[C]=−0.160T+17.408	0.827	35.199	11.744	7	67.7
				15.1	14.978	15	0.14
	818 ($P_{F2}$)	[C]=−0.179T+17.889	0.8461	33.93	11.965	7	70.9
				13.81	15.547	15	3.6
Fructose	870 ($P_{F3}$)	[C]=−0.177T+19.26	0.912	20.458	15.629	7	123.2
				40.426	12.084	15	19.4
	924 ($P_{F4}$)	[C]=−0.192T+21.337	0.974	45.789	12.504	7	78.6
				23.81	16.744	15	11.6
	978 ($P_{F5}$)	[C]=−0.190T+19.296	0.925	31.523	13.281	7	89.7
				2.881	18.746	15	24.9
	832 ($P_{S1}$)	[C]=−0.3141T+23.354	0.8544	39.063	11.084	7	58.3
				35.09	12.33	15	17.8
	868 ($P_{S2}$)	[C]=−0.3283T+23.906	0.8824	41.083	10.418	7	48.8
Sucrose				33.482	12.914	15	13.9
	922 ($P_{S3}$)	[C]=−0.5049T+22.716	0.898	23.976	10.610	7	51.6
				14.902	15.192	15	1.3
	992 ($P_{S4}$)	[C]=−0.5192T+16.479	0.3569	7.765	12.447	7	77.8
				5.623	13.559	15	9.6
